# High‐Performance Water Purification and Synergistic Hydrovoltaic by Avocado Peels Decorated With Carbon Dots

**DOI:** 10.1002/smsc.70284

**Published:** 2026-04-20

**Authors:** Xiaxia Yang, Yutong Chi, Weiwei Shi

**Affiliations:** ^1^ Division of Natural and Applied Sciences Duke Kunshan University Kunshan China

**Keywords:** biomass, carbon dots, hydrovoltaic, seawater desalination, solar evaporation

## Abstract

Interfacial solar evaporation technology is emerging as a promising solution to the freshwater shortage problem. However, the complex manufacturing process and high cost of artificial photothermal materials hinder its widespread application. This work presents a novel solar evaporator (HCAP), distinct from multi‐step methods and carbonized photothermal structures, developed by decorating avocado peels with carbon dots using a one‐step hydrothermal method. The carbonized avocado peel retains the intrinsic porous microstructure, providing excellent channels for water evaporation. Under one solar irradiation, the average evaporation rate of HCAP was 2.79 ± 0.03 kg m^−2^ h^−1^, the water evaporation efficiency was 155.8%, and the photothermal conversion efficiency was 86%. Compared to the control group of bulk water system, the evaporation rate of HCAP increased by approximately fivefold. Furthermore, owing to its numerous orderly channels and micropores, HCAP evaporators can accelerate the transport of ions and generate excellent hydrovoltaic power, with the output performance of 110.2 mV and 10.54 μA, under one‐sun irradiation. During evaporation, salt concentration gradient and light intensity were investigated as key factors affecting high electricity generation. This work provides a simple and sustainable way to use biomass materials for seawater desalination, wastewater purification, and hydrovoltaic production, driven by solar energy.

## Graphical abstract

A biomass‐derived solar evaporator (HCAP) is fabricated by decorating avocado peels with carbon dots via a simple one‐step hydrothermal process. The hierarchical porous structure enables efficient solar water purification while simultaneously generating hydrovoltaic power. Synergistic photothermal heating and ion migration deliver high evaporation performance and sustainable freshwater‐electricity co‐production from low‐cost waste materials.

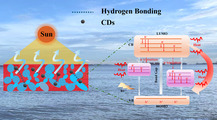

## 1 Introduction

Water is the source of life, the essential component for all living things, and a vital lifeline for modern socioeconomic development [1]. Although nearly 70% of the Earth surface is covered by water, the freshwater necessary for human survival account for only about 2.5% of global water resources [2]. However, most of freshwater exists in the form of glaciers, which are difficult to access directly. As a result, the actual usable freshwater resources for humans account for only 0.26% of the total [3, 4]. At the same time, vigorous development in modern society has led to a series of environmental problems such as global warming and soil erosion [5], coupled with rapid population growth, all of which have resulted in a severe shortage of freshwater [6, 7]. Traditional seawater desalination technologies, such as multi‐effect distillation, multi‐stage flash evaporation, atmospheric distillation, reverse osmosis, and electrodialysis, are insufficient to fully meet the demand [8]. Therefore, solar interfacial water evaporation technology shows great promise for practical applications [9, 10], with advantages such as low heat loss throughout the process, high photothermal conversion efficiency, and low cost [11].

Biomass materials are organic materials extracted from biological organisms such as plants, animals, and microbial derivatives [12, 13, 14]. They are widely available and possess the characteristics of being green, environmentally friendly, low carbon, low cost, renewable, and biodegradable [15]. With the increasing global focus on environmental protection and sustainable energy, they are gradually gaining attention in multiple fields such as energy, materials science, and environmental remediation [16]. When applied to solar interfacial water evaporation technology, these materials not only rely on the unique microchannel structure of natural biomass, exhibiting advantages such as high water transport, high light absorption, and stable freshwater production [17], but also the inherent solar energy utilization and water transpiration characteristics of plants to construct various photothermal structures [18]. Besides the treated biomass, various novel materials with dominant interaction mechanisms have been proven to achieve efficient photothermal conversion. In the field of solar interfacial evaporation research, carbon materials are the most widely used, with typical examples including graphene [19], carbon nanotubes [20], carbon black [21], graphdiyne [22], and biochar [23]. Their photothermal conversion efficiency stems from the lattice vibrations of the carbon framework [24]. Notably, carbon dots (CDs) are increasing attention in the field of solar evaporation due to their broadly spectrum light absorption characteristics, flexible surface functionalization potential, and excellent photothermal conversion efficiency [25, 26].

Water molecules interact physically or chemically with material interfaces, which inevitably leads to changes in energy density during phase transitions. Leveraging this interaction, a new technology called hydrovoltaic technology has been proposed to convert gas–liquid–solid interactions into electrical energy [27]. Recent studies have demonstrated various strategies for simultaneous freshwater and electricity generation. S.Y et al. fabricated a MIL‐53Al hydroelectric generator that achieved a high open‐circuit voltage (270 mV) and short‐circuit current (14 μA) in seawater [28]. L.K et al. developed a dual‐drive water evaporation generator (DWEG) that continuously generates a high voltage of 1.13 V and a stable current of 10.54 µA (1.76 µA cm^−2^) under ambient conditions (approximately 20°C and 43% Relative Humidity) [29]. H.G et al. designed a low‐cost carbon foam‐based bifunctional evaporator that achieves the voltage of 0.33 V and current of 14.4 µA output. In conjunction with an evaporator, this integrated technology produces both clean water and electricity [30]. Z. K et al. prepared a nanocomposite cellulose membrane, which achieved a high output voltage of approximately 3.7 V [31]. The hydrovolt voltage has grown rapidly from the initial millivolt level to the volt level in just a few years, demonstrating enormous development potential [27]. While these systems achieve impressive performance, they typically rely on multi‐component device integration, additional thermoelectric modules, or relatively complex fabrication routes.

In contrast, our evaporator integrates photothermal evaporation and hydrovoltaic electricity generation within a single biomass‐derived material, offering a simpler architecture and potentially lower cost. Here, this evaporator employs a one‐step hydrothermal method to decorate avocado peel (AP) with carbon dots (CDs), called HCAP. In this process, the avocado peel itself not only serves as a substrate but also provides carbon source, thus achieving the photothermal performance in one step. The carbonized avocado peel retains the porous hierarchical structures, common in biomass, providing excellent channels for water transport and evaporation. Under one solar irradiation, the water evaporation rate of HCAP was 2.79 ± 0.03 kg m^−2^ h^−1^, five times better compared to the control group of bulk water, and the water evaporation efficiency was 155.8%. The HCAP also demonstrates long‐term durability and the excellent ability to purify seawater and wastewater containing various impurities. Notably, the HCAP can achieve synchronous power generation through the synergistic effect of water voltaic and salt diffusion, achieving optimal results in the saline solution. Furthermore, the hydrovoltaic efficiency increases with increasing light intensity, and the salt concentration gradient can serve as a key factor for power harvesting. These findings contribute valuable insights into the simple synthesis and practical applications of decorative biomass materials as solar interfacial evaporators, including seawater desalination, wastewater treatment, and hydrovoltaic production.

## 2 Results and Discussion

**FIGURE 1:**
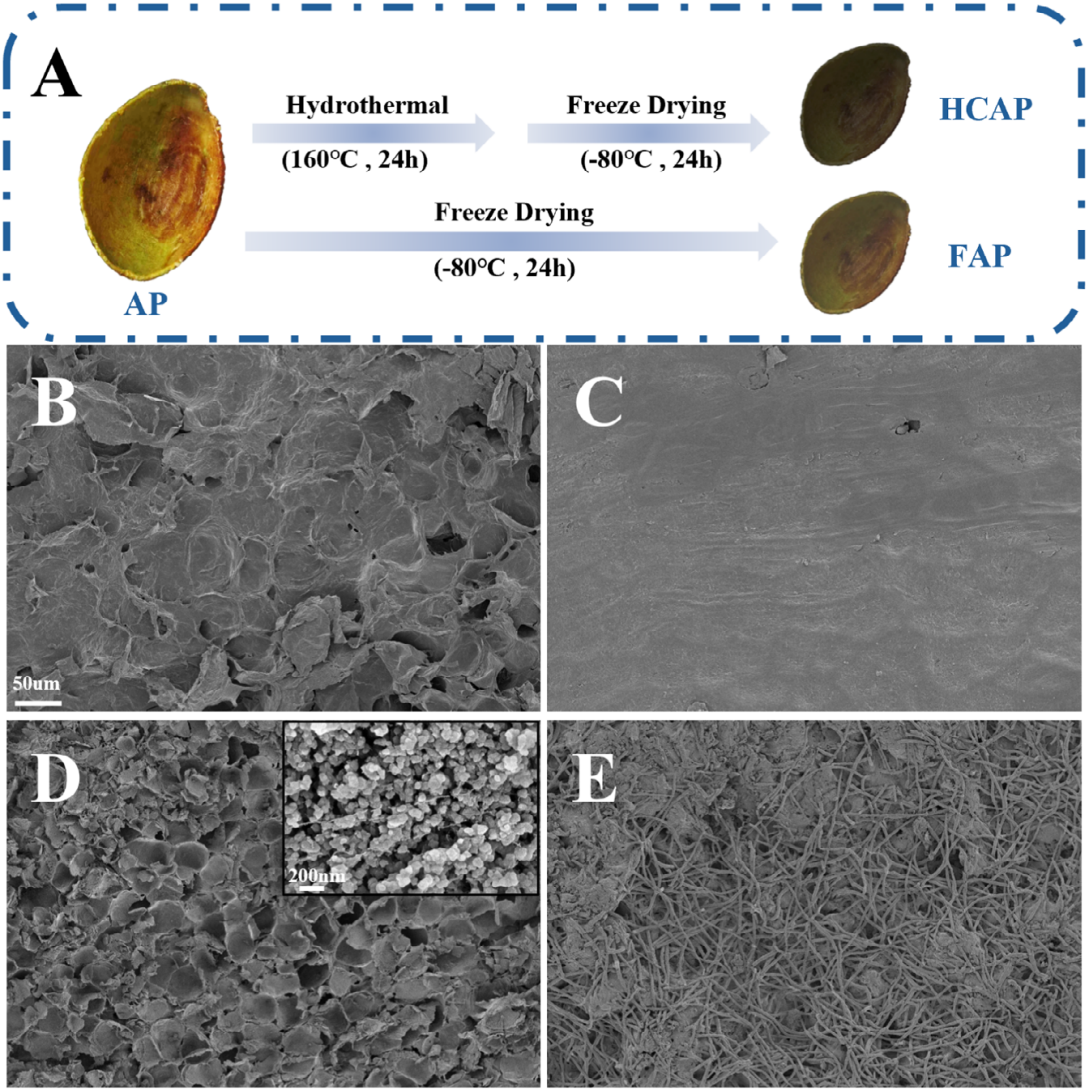
(A) Schematic of the preparation procedures of FAP and HCAP. The SEM images of (B) FAP top, (C) FAP bottom, (D) HCAP top, and (E) HCAP bottom, respectively. The inset shows the nanoparticle structures of carbon dots (CDs).

Figure 1A presents the fabrication process of the bio‐based evaporators. First, the pristine avocado peel (AP) was converted into freeze‐dried avocado peel (FAP) via freeze‐drying. The hydrothermal carbonized avocado peel (HCAP) was prepared with the carbon dots (CDs) loaded on FAP through the simple one‐step process, and then freeze‐drying. To further investigate the bio‐inspired evaporators based on the avocado peel, scanning electron microscopy (SEM) was used to observe the top and bottom surfaces of the FAP and HCAP, as these morphologies directly affect their solar interfacial evaporation performance. The top surface of the FAP (Figure 1B) exhibits the porous honeycomb structure characteristic of natural biomass, presenting a layered, loosely stacked structure with irregular micropores. These pores are composed of interlaced cell walls, with a relatively rough surface and attached minute natural textures, exhibiting superhydrophilicity (Figure S1A). In contrast, the bottom surface of the FAP (Figure 1C) exhibits a dense and plain structure, and the special wettability shows an average water contact angle of 72.8° ± 1.3° in 120s (Figure S1C).

After hydrothermal carbonization, the microstructure of the HCAP changed significantly. The top surface of the HCAP (Figure 1D) retained a honeycomb‐like porous structure similar to that of the FAP, indicating that the hydrothermal process did not damage the mechanical stability of the avocado peel skeleton. This hierarchical porous structure provides interconnected capillary channels for water transport from the bulk to the surface, maintaining an efficient water transport network [32]. The SEM image in Figure 1E demonstrates the most significant change occurred at the bottom surface of the HCAP. Unlike the FAP, the surface of the HCAP exhibited an intertwined fibrous network structure, with the diameters ranging from 2.09 to 4.66 μm. Both the top and bottom surfaces of HCAP were superhydrophilic, with the water drop spreading entirely in 0.26 and 0.38 s (Figure S1B and S1D), respectively, thus maintaining a continuous water supply to the evaporation surface. The high‐magnification SEM image (Figure 1D, inset) clearly show that a large number of uniformly sized CDs particles are tightly and evenly loaded on the HCAP, with a diameter of 61.65 nm, approximately. These unique nanoparticles enhance light capture from sun irradiation for solar water evaporation. The composite of the carbonized black skeleton and carbon dots significantly improves wide spectrum absorption, while light reflection is reduced through multiple scattering induced by the nanostructure. Furthermore, it increases the effective contact area at the water‐air interface and creates numerous open microchannels, facilitating rapid desorption and diffusion of water vapor [33].

**FIGURE 2:**
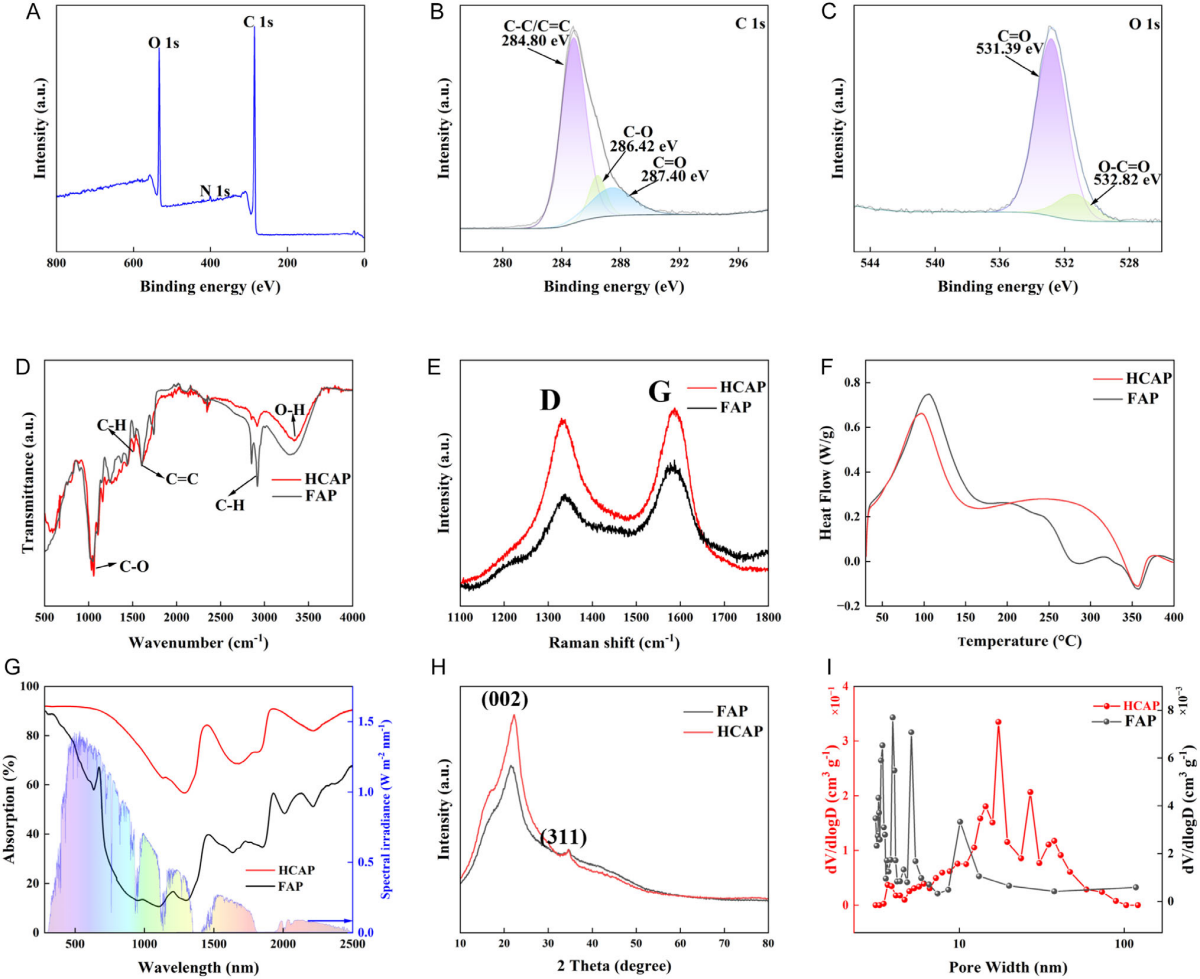
(A) The full spectrum scan of XPS spectra, (B) the C 1s core‐level spectra, and (C) O 1s core‐level spectra of HCAP. (D) The FT‐IR spectra, (E) Raman spectra, (F) DSC heating curves, (G) UV–Vis–NIR spectra over the wavelength ranges of 280–2500 nm, (H) XRD spectra, and (I) pore size distribution of FAP and HCAP, respectively.

This study employed X‐ray photoelectron spectroscopy (XPS) and Fourier transform infrared spectroscopy (FT‐IR) to investigate the chemical properties of FAP and HCAP. Figure 2A demonstrates the full‐spectrum scan of HCAP, which clearly revealed the main peaks of C 1s and O 1s, as well as N 1s, consistent with the carbon‐based framework of hydrothermally carbonized biomass and CDs [34, 35]. In the C 1s core‐level spectrum (Figure 2B), peaks corresponding to C—C(aliphatic)/C=C(aromatic), C—O, and C=O were observed, confirming the successful integration of CDs and the presence of oxygen‐containing functional groups. The O 1s core‐level spectrum in Figure 2C further subdivided into C=O and C—O bonds; these hydrophilic functional groups contribute to special wettability of the material during solar interfacial evaporation [36]. Furthermore, the N 1s core‐level spectrum (Figure S2) shows a peak at 400.17 eV, which corresponds to the C—N—C bond in HCAP.

The FT‐IR spectra in Figure 2D further distinguished FAP from HCAP. Both FAP and HCAP exhibited strong peaks near ~3330 cm^−1^, attributed to the O—H stretching vibrations of cellulose and hemicellulose [37]. A strong C—O stretching vibration peak is observed at ~1050 cm^−1^, a characteristic peak of natural biomass. In contrast, HCAP shows a weakened C—H peak at ~2920 cm^−1^, while the C=C peak at ~1620 cm^−1^ and the C—H peak at ~1500 cm^−1^ are enhanced [1]. This is consistent with XPS results, confirming that dehydration and decarboxylation reactions occurred in the avocado peel during hydrothermal carbonization, forming a more stable carbon framework. Figure 2E shows the Raman spectra which revealed the degree of graphitization in FAP and HCAP. The D peak at 1334 cm^−1^ corresponds to defect‐induced vibrations. The G peak at 1586 cm^−1^ corresponds to the in‐plane C=C stretching vibration of sp^2^ hybridized carbon atoms [35]. At a 532 nm light source, the calculated intensity ratio (*I*_D_/*I*_G_) of HCAP is 1, while that of FAP is 0.94, indicating that the HCAP is in the advanced disordered state with more pronounced graphitization behaviors. This suggests that HCAP simultaneously possesses characteristics of disordered and ordered carbon, synergistically enhancing solar absorption by increasing photon trapping, electronic density of states, and thermal conductivity, ultimately improving photothermal performance. Differential scanning calorimetry (DSC) characterized the thermal behavior as shown in Figure 2F. FAP and HCAP showed similar peak structures but there is a peak at 282°C in FAP while no peak in HCAP, indicating the exfoliation of lignin during hydrothermal processes. The peak at 105°C is attributed to an endothermic peak caused by the evaporation of volatile water molecules or bound water [38]. No endothermic or exothermic peaks were observed in the range of 105°C–358°C, indicating the stability of HCAP within this temperature range. The presence of an exothermic peak or melting point at 358°C suggests the thermal decomposition of the cellulose and starch chains [39]. Multiple decompositions in the 194°C–334°C range are attributed to the degradation of hemicellulose, lignin, and cellulose [40]. The degradation temperature range of lignin is wider than that of cellulose and hemicellulose, attributed to its presence of functional groups such as phenolic hydroxyl, hydroxyl, carboxyl, carbonyl, and methoxy groups.

Figure 2G demonstrates the UV–Vis–NIR absorption spectra of FAP and HCAP. The FAP exhibits a low absorbance of 49.95% in the range of 280–2500 nm. In contrast, HCAP demonstrates a higher absorbance of 83.68% across the entire solar spectrum, approximately twice that of FAP. This is attributed to the enhanced broadband absorption and improved light utilization due to the carbon‐rich framework and carbon dots after the simple hydrothermal treatment. The structural characteristics of FAP and HCAP were further investigated using X‐ray diffraction (XRD), as shown in Figure 2H. The XRD patterns of both FAP and HCAP exhibited broad diffraction peaks centered around ~22°, corresponding to the crystal plane (002) of graphitic carbon [41]. A low‐intensity diffraction peak at 34.5° in FAP and HCAP, corresponding to the mixed inorganic components in the avocado peel, crystal plane (311) [42]. The full width at half maximum (FWHM) of the peak (002) in HCAP is 10.2°, which indicates the presence of turbostratic or amorphous carbon domains with limited long‐range order, commonly observed in carbon materials extracted from lignocellulosic biomass [41, 43]. The heterogeneous structure facilitates solar evaporation through multiple scattering of light and thermal conduction of graphitized microcrystals within the disordered regions [44]. The peak (002) in HCAP is higher than that of FAP, which indicates the presence of carbon dots in HCAP [45]. The pore size distribution of the FAP and HCAP were systematically studied using nitrogen physically adsorption and desorption isotherms, as shown in Figure 2I. FAP exhibits a narrow pore size distribution in the small mesoporous region (2–10 nm), indicating the presence of a well‐structured but limited mesoporous network in FAP. The hysteresis loop of the adsorption and desorption curve in Figure S3A also reflects the characteristics of this narrow, slit‐like pore structure of mesoporous materials. In stark contrast, HCAP exhibits a wider pore size distribution in the range of 10–20 nm, in addition to contributions from larger mesopores (20–50 nm) and macropores (>50 nm). In Figure S3B, each HCAP shows a type IV isotherm with a distinct H3 hysteresis loop, indicating a uniform macroporous structure. Furthermore, the specific surface area of HCAP (21.16 m^2^g^−1^) via BET is ≈9 times that of FAP (2.22 m^2^g^−1^). The hierarchically porous structures of HCAP enable efficient water transport from the reservoir to interface and promotes thermal localization, resulting in an excellent evaporation rate.

High‐resolution transmission electron microscopy (HR‐TEM) was employed to further characterize the carbon dots, as shown in Figure S4. The positions of the rings in the SAED pattern (Figure S4A), agree well with the standard diffraction data for graphitic carbon, corresponding to the (111), (220), and (311) crystal planes of graphite. This confirms that the CDs possess a graphitic structure. The TEM images shown in Figure S4B and C show that the CDs are uniformly dispersed without obvious agglomeration, indicating their excellent stability. The inset displays the uniform particle sizes of CDs, which are mainly distributed in the range of 1–3 nm with an average size of 1.99 ± 0.94 nm. The spherical morphology remains well maintained throughout the field of view, without any visible aggregation, demonstrating that the hydrothermal carbonization method can effectively prepare monodisperse nanoscale particles. Clear lattice fringes are observed in the HR‐TEM image in Figure S4D with a lattice spacing of 0.1991 nm, attributed to dehydration and aromatization during hydrothermal treatment, which is very close to the in‐plane lattice spacing (100) of graphene [40]. This confirmed the crystalline nature of the CDs, which is consistent with graphitic carbon domains. These structural features are beneficial for efficient light absorption and photothermal conversion.

**FIGURE 3:**
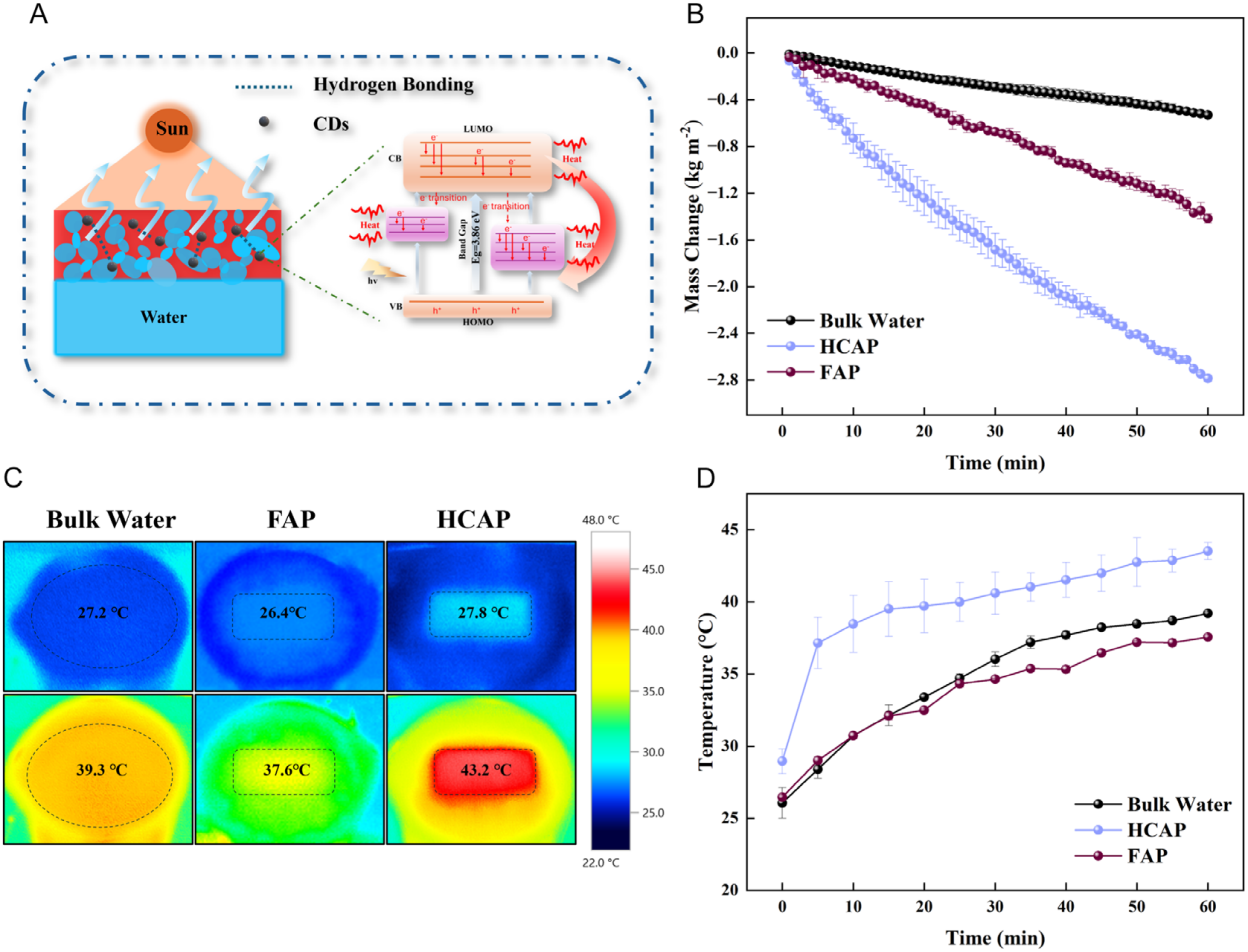
(A) Photothermal and water transport mechanisms of HCAP solar steam generation unit. (B) Mass variation of HCAP, FAP, and bulk water under one‐sun irradiation. (C) The thermal IR images of HCAP, FAP, and bulk water at the initial stage of 0 min and the final stage of 60 min. (D) Temperature variation of HCAP, FAP, and bulk water during the solar evaporation process.

The solar interfacial evaporation performance of HCAP was evaluated in Figure 3A. The HCAP evaporator was irradiated using a solar simulator, and mass changes were monitored accordingly. When exposed to solar irradiation, CDs effectively absorb solar energy across a broad spectrum from ultraviolet to the first near‐infrared window [46]. The photothermal conversion process of HCAP is primarily governed by the electronic transitions and relaxation behavior of CDs. Upon solar irradiation, the CDs exhibit broadband light absorption and undergo electronic excitation from the highest occupied molecular orbital (HOMO) to the lowest unoccupied molecular orbital (LUMO). In this case, the electron is likely excited from the π to π* orbital since the π bonds have a lower energy gap from their weaker bond compared to the σ‐bond [47]. The CDs’ bandgap (*E*_g_) was estimated by drawing a tangent at the steepest slope in the region via the Tauc plot method, as demonstrated in Figure S5A. The value at the intersect of tangent with the *x*‐axis was 3.86 eV, derived from UV–Vis absorption and Tauc plot analysis. The excited charge carriers rapidly relax back to the ground state predominantly through non‐radiative pathways rather than radiative emission. This non‐radiative relaxation process converts the absorbed photon energy into thermal energy via strong electron‐phonon coupling [48].

The presence of multiple states between the LUMO and HOMO energy levels, introduced by various surface chemical groups, leads to enhanced nonradiative relaxation and fluorescence quenching due to the small band gap and various surface states containing nitrogen‐related functional groups [49, 50]. The generated heat is efficiently localized at the evaporation interface owing to the intimate contact between the carbon dots and the porous avocado‐peel matrix. This localized thermal energy is subsequently transferred to the interfacial water, accelerating water evaporation. The combination of efficient photon absorption, suppressed radiative loss, and effective heat localization accounts for the high photothermal conversion capability of HCAP under solar irradiation [51]. Therefore, CDs will be beneficial for the conversion of solar energy into thermal energy, a type of end‐use energy [52]. This property ensures efficient solar‐thermal energy conversion during interfacial evaporation, with the photothermal conversion efficiency of 86% on HCAP (Supporting Information Note S1).

Figure 3B shows the mass changes of HCAP, FAP, and bulk water over 60 min solar evaporation under one‐sun irradiation. The average evaporation rate of HCAP was 2.79 ± 0.03 kg m^−2^ h^−1^, approximately 5 times higher than that of bulk water, that is 0.53 ± 0.03 kg m^−2^. The average evaporation rate for FAP was 1.42 ± 0.04 kg m^−2^ h^−1^, which is about 2.6 times that of bulk water but lower than that of HCAP. This high evaporation rates of HCAP attribute to the synergistic effect: on one hand, the carbon‐rich framework (sp^2^ carbon) and CDs can absorb most of solar energy and convert it into thermal energy through non‐radiative relaxation; On the other hand, the hierarchical porous structure promotes a continuous supply of water to the high‐temperature interface through capillary action, ensuring uninterrupted water evaporation and preventing interface drying. The control comparison of the HCAP evaporator performance with and without Styrofoam was investigated under otherwise identical conditions. The average evaporation rate was 2.39 ± 0.01 kg m^−2^ h^−1^ (Figure S6A), with final temperatures of 42.33°C ± 0.55°C (Figure S6B) and the water evaporation efficiencies was calculated as 130.5%. The evaporation performance of HCAP without Styrofoam is less than that of HCAP with Styrofoam, but better than that of FAP. This directly demonstrates the influence of insulation and confirms that the performance enhancement of HCAP remains evident even without Styrofoam, while Styrofoam primarily improves test fidelity by reducing background evaporation from the surrounding water surface.

The temperature changes in Figure 3C,D further confirmed this behavior. Note that the temperature at 0 min represents the first recorded value immediately after illumination onset rather than a fully equilibrated baseline. Minor differences at 0 min arise from data acquisition timing and do not affect the steady‐state temperature comparison. Throughout the test, the temperature of the bulk water rose slowly from 26.1°C ± 1.1°C to 39.2°C ± 0.1°C, and remained below that of the HCAP. The surface temperature of the HCAP increased to approximately 43.2°C ± 0.6°C within 60 min and then stabilized. In contrast, the surface temperature of FAP slowly increased from 26.4°C ± 0.2°C to 37.6 ± 0.2°C at 60 min, better than that of the bulk water but less than that of HCAP. This heat absorption by the evaporator alone prevents the bulk water from heating up, thereby maximizing the energy utilization of surface evaporation. The thermal infrared (IR) images (Figure 3C) visualize the temperature distribution on the evaporation surfaces. The temperature without HCAP was lower than that of the surface with HCAP, and the temperature distribution was uniform. This localized heating confirms that the HCAP can confine photo‐generated heat to the evaporation interface, which is crucial for minimizing heat loss and maximizing evaporation efficiency. Figure S5B shows the temperature increase on HCAP, FAP, and bulk water under one solar irradiation. During the solar evaporation process, the surface temperature of HCAP, FAP, and bulk water increased by 8.2 ± 1.0°C, 2.5 ± 0.3°C, and 2.3 ± 0.6°C, respectively, in the initial 5 min. In the final stage of 60 min, the temperature difference reached 14.6 ± 1.3°C, 11.1 ± 0.3°C, and 13.1 ± 1.0°C, corresponding to HCAP, FAP, and bulk water, respectively. This clearly demonstrates the performance enhancement achieved by carbon dot decoration on HCAP relative to both bulk water and FAP.

**FIGURE 4:**
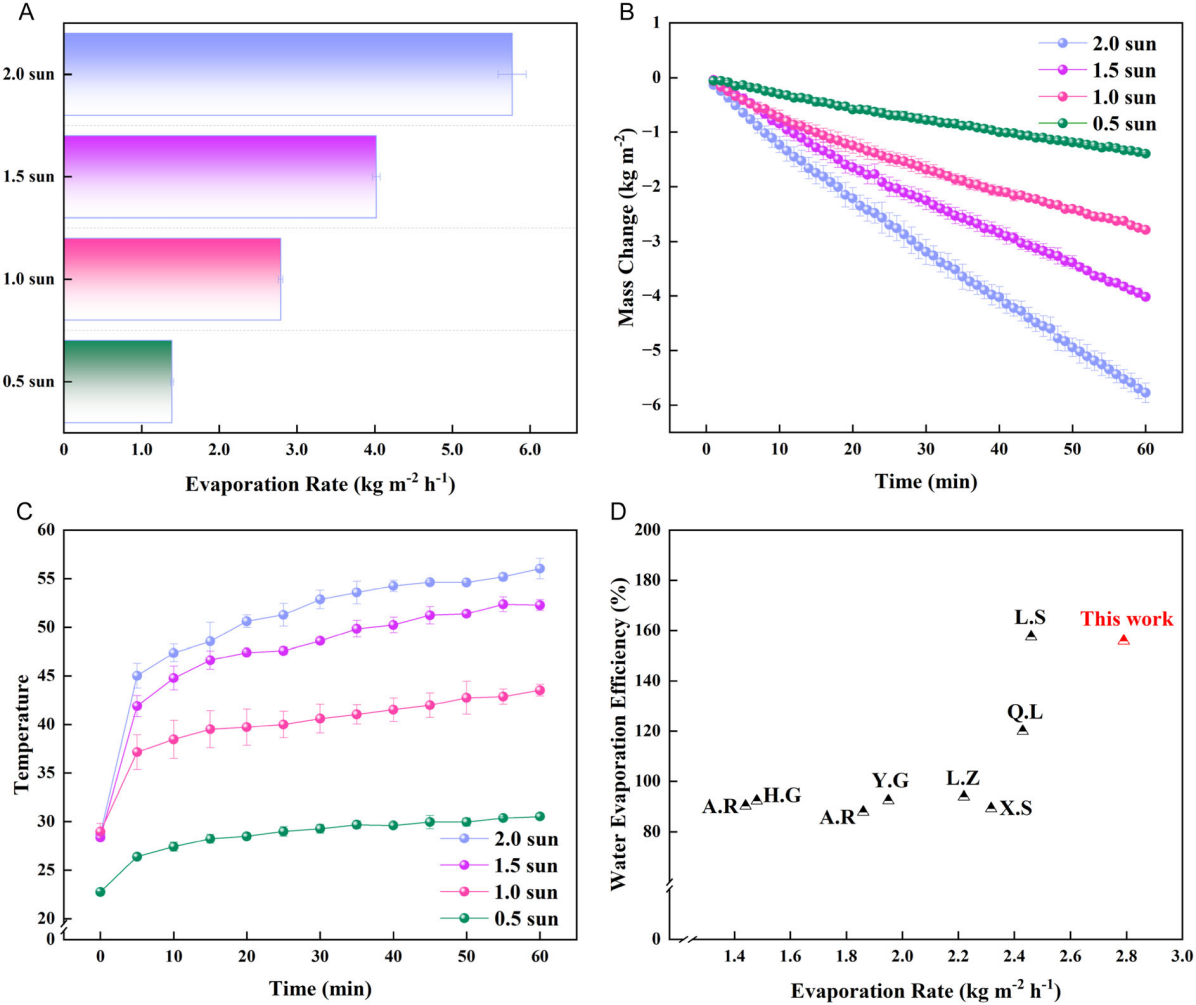
(A) Evaporation rates of HCAP at different solar intensities of 0.5‐sun, 1.0‐sun, 1.5‐sun, and 2.0‐sun irradiation, respectively. (B) Mass changes of HCAP and temperature variations at different solar intensities. (C) Surface temperature of HCAP at different solar intensities of 0.5‐sun, 1.0‐sun, 1.5‐sun, and 2.0‐sun irradiation, respectively. (D) The comparison of various solar interfacial evaporators under one‐sun irradiation.

To evaluate the adaptability of the HCAP evaporator at different solar irradiation intensities, Figure 4A quantifies the steady‐state evaporation rates of the HCAP. As the solar irradiation intensity increases from 0.5 sun to 2.0 sun, the evaporation rate varies nearly linear and increases over four times higher, from approximately 1.39 ± 0.02  to 5.77  ± 0.18 kg m^−2^ h^−1^. This positive correlation results from the efficient solar thermal energy conversion and minimal heat loss on HCAP. This indicates that higher irradiation intensity transfers more energy to the interface and accelerates water evaporation. The mass changes in Figure 4B further reflects more details in this trend, with the fastest mass loss observed in HCAP at 2.0 sun and the slowest at 0.5 sun. The linear mass change over time confirms the stable water transport and evaporation performance of HCAP. Correspondingly, Figure 4C shows that the steady‐state surface temperature distribution on HCAP, which increases with increasing solar irradiation intensity, at most 30.54 ± 0.30°C, 43.52 ± 0.60°C, 52.28 ± 0.54°C, and 56.03 ± 1.07°C, corresponding to 0.5 sun, 1.0 sun, 1.5 sun and 2.0 sun, respectively. This temperature increase directly drives a higher evaporation rate without excessive overheating, indicating that the low thermal conductivity of HCAP effectively confines heat to the interface, avoiding bulk water heating and energy waste. To better understand the performance of HCAP, Figure 4D compares its evaporation rate and water evaporation efficiency at 1.0 sun irradiation with other biomass‐derived interfacial evaporation materials [2, 41, 53, 54, 55, 56, 57, 58]. The HCAP has an evaporation rate of 2.79 ± 0.03 kg m^−2^ h^−1^, and the water evaporation efficiency of 155.8% (Supporting Information Note S2), outperformed the previous work. This water evaporation efficiency of 155.8% is likely because the sidewall temperature of 21°C is lower than the ambient temperature of 30°C, leading to net heat gain from the environment via convection and radiation, which, according to Newton's law of cooling, promotes water evaporation, resulting in an evaporation efficiency of over 100% [59, 60]. This is verified by the detailed calculations in the Supporting Information Note S3.

**FIGURE 5:**
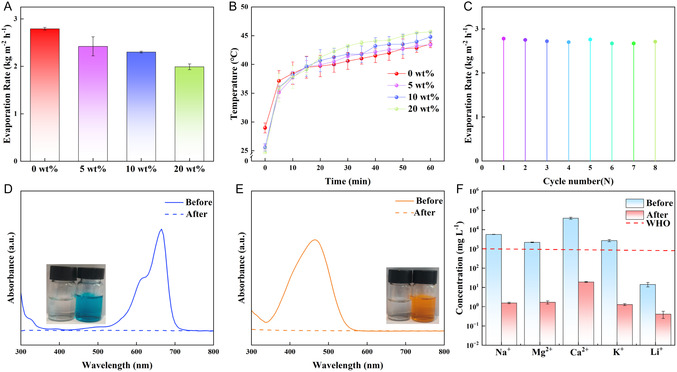
(A) Evaporation rates for HCAP at different salt concentration of 0wt%, 5wt%, 10wt%, and 20wt%, respectively. (B) Temperature profiles for HCAP at different salt concentration. (C) The long‐term tests of HCAP in 8 cycles under one‐sun irradiation. (D,E) UV–Vis spectra of different dyes, methylene blue (MB), and methyl orange (MO), before and after purification, with the corresponding photos as insets. (F) Ion concentration in real seawater (Yellow Sea, China) before and after desalination.

To verify the practical applications of HCAP in seawater desalination and wastewater treatment, we tested various performance including high salinity conditions, cyclability, and pollutant removal capacity. Figure 5A presents the evaporation rates of HCAP in different saline waters. HCAP maintains a stable evaporation performance (Figure S7A), with the average rates of 2.79 ± 0.03, 2.42 ± 0.20, 2.30 ± 0.02, and 1.99 ± 0.06 kg m^–2^ h^–1^, corresponding to 0wt%, 5wt%, 10wt%, and 20 wt% NaCl solutions, respectively. This excellent salt resistance is owing to the hierarchically porous structures of HCAP, where interconnected microchannels facilitate continuous water transport, while the fibrous skeleton confines crystalline salts within the pores. As the NaCl concentration increases, the surface temperature rise becomes more pronounced (Figure 5B). This trend can be attributed to two coupled factors. First, dissolved salt lowers the equilibrium vapor pressure of water (solute effect), which suppresses the evaporation flux and thus weakens evaporative cooling; consequently, a larger fraction of absorbed energy contributes to sensible heating and elevates the surface temperature. Second, the specific heat capacity of saline solutions decreases with increasing concentration, so a given heat input produces a larger temperature increase compared to pure water. Together, suppressed evaporative cooling and reduced heat capacity account for the higher observed temperatures at higher salinities under identical irradiation conditions.

Figure 5C evaluates the cyclability of HCAP over eight consecutive evaporation cycles, in total 8 h. The evaporation rate remained around 2.70 kg m^−2^ h^−1^ with no significant fluctuations. Figure S7B shows an optical photograph after the eight cycles, with no obvious salt accumulation on the surface. To evaluate the salt‐resistance and structural stability of HCAP during cyclic desalination, SEM characterization was performed after 8 evaporation cycles. As shown in Figure S8, the post‐cycling HCAP retains its interconnected porous architecture and clear fibrous structures, without detectable salt deposition or pore blockage, which is consistent with the stable evaporation performance observed during repeated operation. To further verify the salt‐resistance, EDS analysis was conducted on the HCAP surfaces before and after 8 desalination cycles. As shown in Figure S9, Na and Cl signals are negligible both before and after cycling, indicating that no salt accumulation occurs on the HCAP surface during repeated operation. Combined with SEM observations, these results demonstrate that HCAP maintains its structural integrity and effective salt‐rejection capability under cyclic desalination conditions.

The stability of performance after eight cycles indicates that HCAP has the potential for long‐term practical application. The wastewater purification capacity of HCAP was tested using the cationic dye methylene blue (MB) and the anionic dye methyl orange (MO), as well as real seawater (Yellow Sea, China). UV–Vis spectroscopy in Figure 5D,E demonstrated that there are obvious absorption peaks from the MB‐ and MO‐contaminated solutions, while the peaks disappeared in the water collected from the solar evaporation process. The carbonaceous framework of HCAP adsorbs the dye onto the fiber surface, while the evaporation process separates pure water vapor from the contaminants. The inset visually confirms the colorless, with the condensed water appearing clear. Figure 5F revealed the various ion concentrations before and after the real seawater desalination process, characterized by ICP‐MS. The ion concentration of Na^+^, Mg^2+^, Ca^2+^, K^+^, and Li^+^, after desalination, is far below the drinking water standards, defined by the World Health Organization [61]. The salt rejection ratio achieved 99.97%, calculated from the equation in Supporting Information Note S4. This high performance is attributed to the interfacial evaporation mechanism, where only water molecules evaporate, while ions are trapped in the bulk solution or adsorbed onto the HCAP framework.

**FIGURE 6:**
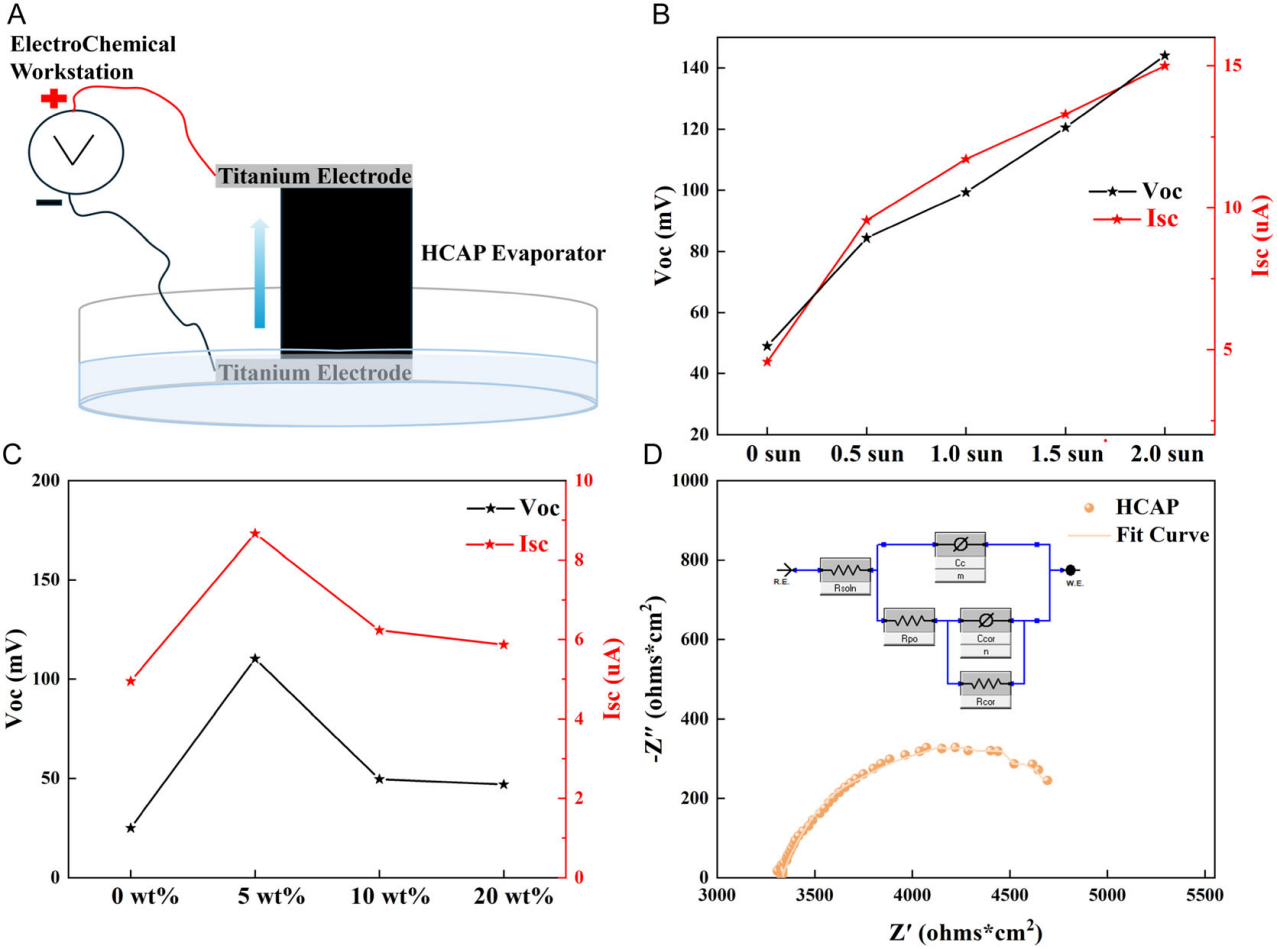
(A) The schematic diagram of the hydrovoltaic generation setup. (B) The output performance of HCAP under different solar fluxes. (C) The output performance of HCAP in different concentrations of saline water under one‐sun irradiation. (D) Nyquist diagram of the HCAP.

Figure 6A shows a typical experimental setup for evaporative electricity generation, where two electrodes are connected to the top and bottom of the HCAP. The solution of 5wt% NaCl is added to the container so that it contacts the bottom of the sample, meaning the bottom electrode is wet while the top electrode remains dry in the air. It should be emphasized that titanium electrodes were chosen during the experiment to mitigate the influence of corrosion potential [62]. Figure 6B measures the changes in the open circuit voltage (*V*_oc_) and short‐circuit current (*I*_sc_) of the HCAP with different solar irradiation intensity. Both the open‐circuit voltage and short‐circuit current show a continuous enhanced trend with increasing solar fluxes. As the irradiation varies from 0 sun, 0.5 sun, 1.0 sun, 1.5 sun, to 2.0 sun, the open‐circuit voltage reaches 43.79, 90.13, 110.2, 124.9, and 140.7 mV, and the short‐circuit current achieves 5.12, 8.93, 10.54, 12.83, and 15.36 μA, respectively. The enhanced output performance under illumination can be attributed to the interaction of local fields [63]. Stronger illumination accelerates water evaporation, thereby enhancing the ion concentration gradient and migration dynamics between the electrodes, and thus promoting the generation of electrical signals. Although the absolute electrical output is lower than that of thermoelectric‐assisted systems, the HCAP platform provides a sustainable and scalable route for decentralized freshwater‐electricity co‐production.

Figure 6C depicts the output performance of the HCAP in different concentrations of NaCl solution under one solar irradiation. As the concentration increased from 0 wt% to 20 wt%, *V*_oc_ and *I*_sc_ peaked at 5 wt%, achieving 110.2 mV and 8.67 uA, respectively, likely due to the shortening of the Debye length [63]. The optimal performance at 5 wt% NaCl solution, close to the salinity of real seawater, indicates that the HCAP device is feasible for practical applications. The Nyquist plot obtained in Figure 6D by equivalent circuit further reveals the electrochemical impedance characteristics of theHCAP. To quantitatively analyze the EIS data, a modified equivalent circuit was constructed, as illustrated in the inset of the figure, and used to fit the experimental impedance data. The Nyquist plot exhibits a characteristic single semicircle in the high‐to‐medium frequency region, followed by a slight decline at low frequencies. This feature of well‐defined semicircle in the medium‐frequency range is primarily attributed to the charge transfer process, with its diameter directly corresponds to charge transfer resistance (*R*_cor_). The slight downward deviation of the curve at low frequencies is, due to the porous structure of biomaterials. The corrosion resistance *R*_cor_ is 407.5 ohms cm^2^, which facilitates the generation of electricity from biomaterials.

Compared with previously reported freshwater‐electricity co‐generation systems, the present HCAP evaporator offers several distinct advantages. First, HCAP is derived from abundant waste biomass (avocado peels) and fabricated via a simple one‐step hydrothermal process, avoiding complex multi‐component assembly or high‐temperature carbonization. Second, the hierarchical porous structure and carbon‐dot decoration enable efficient solar absorption, localized thermal management, and continuous water transport, resulting in a high evaporation rate of 2.79 kg m^−2^ h^−1^ under one‐sun irradiation. Third, the integrated hydrovoltaic effect allows simultaneous electricity generation driven by water evaporation and ion migration, without the need for external thermoelectric modules or additional energy‐conversion units. These features collectively highlight the simplicity, sustainability, and multifunctionality of the HCAP system.

## 3 Conclusion

In summary, a solar interfacial evaporator was fabricated by modifying avocado peel with carbon dots using a one‐step hydrothermal method. The solar evaporation rate was 2.79 ± 0.03 kg m^−2^ h^−1^, approximately five times higher than that of a bulk water system, the water evaporation efficiency achieved as high as 155.8%**,** and the photothermal conversion efficiency was 86%. The excellent performance is attributed to the ideal photothermal conversion capacity and hierarchically porous structures of the carbon dots modified on the avocado peel, which achieves a good balance between capillary flow and insulation. This ensures sufficient water transport during solar evaporation and effective insulation to prevent heat loss. HCAP showed excellent self‐cleaning performance, good stability, and durability for long‐term applications. Furthermore, HCAP can generate excellent hydrovoltaic power, with the open‐circuit voltage of 110.2 mV and the short‐circuit current of 10.54 μA, under one‐sun irradiation, where solar irradiation intensity and salt concentration gradient are key factors affecting high electricity output. This study provides a simple method to achieve the dual functions of water purification and hydrovoltaic in only one platform, feasible for recovering biomass materials using solar energy.

## 4 Experimental Section

### 4.1 Materials

The avocado fruits were purchased from Hema Supermarket in Suzhou, China. Glucose and sodium chloride were purchased from Sigma Pharmaceuticals & Chemicals. Methylene blue and methyl orange were purchased from Titan Technologies. Seawater was collected from the Yellow Sea, China.

### 4.2 Material Preparation

Washed the pristine avocado peels (AP) with DI water, and then immersed them in the polytetrafluoroethylene (PTFE) reactor containing the 8wt% glucose solution. The reaction conditions are 160°C for 24 h. After the reaction was complete, washed again with DI water, and then froze them at −80°C for 4 h. Afterwards, placed them in a freezer, freeze‐drying for 24 h to obtain HCAP. As the comparison, FAP is freeze‐dried immediately after the first washing step.

### 4.3 Material Characterization

Structures were analyzed by Empyrean X‐ray diffractometry (XRD; Rigaku Ultima IV, Japan). Chemical bonding was studied by using X‐ray photoelectron spectroscopy (XPS; Thermo Fisher Scientific, USA). Surface morphology was examined under a scanning electron microscope (SEM; Thermo Fisher Scientific, USA). The elemental composition and distribution of the sample were characterized by energy‐dispersive X‐ray spectroscopy (EDS**;** TESCAN VEGA4**;** Czech). Surface functional groups were identified via Fourier transform infrared spectrometry (FT‐IR; INVENIO; Bruker, Germany). Raman spectroscopy was employed using a laser Raman microscope (LabRAM HR Evolution, Horiba, Japan). Solar absorption and band gap was assessed with a UV–Vis–NIR spectrophotometer (Cary 5000; USA). UV–Vis spectroscopy was used to characterize the adsorption process of organic pollutants (Agilent Cary 60; USA)**.** Contact angles were measured with a dedicated meter (OCA25; DataPhysics, Germany). Differential scanning calorimetry was performed using Discovery 25 (DSC; TA Instruments, USA). Nitrogen physical adsorption‐desorption test was performed using (BET; Anton Paar Autosorb 6100, USA) instruments, and the pore size distribution was modeled by BJH. Ion concentrations were obtained by plasma mass spectrometry analysis (ICP‐MS; Agilent 7850, USA). The morphology and the selected area electron diffraction of the CDs were characterized by transmission electron microscopy (TEM; JEM‐F200; Japan).

### 4.4 Evaporation Experiments

Under a solar simulator (Fode Lighting Equipment, Shanghai) with high irradiation uniformity, the evaporation system including HCAP or FAP, was set up on a digital mass balance (AX1502ZH, OHAUS). The digital mass balance recorded the mass change every 1 minute. A digital solar power meter (Fode Lighting Equipment, Shanghai) was used to measure the solar simulator's light intensity. In a typical evaporation test, 80 mL of water was added into the beaker. The vertical distance between the xenon lamp and the water surface was fixed at 80 cm for all experiments. The solar irradiation area is about 0.084 m^2^, which ensures the intensity of light all over the evaporative surfaces is always 1 kW/m^2^, confirmed by measurements in the experiments, via the digital solar power meter. Infrared thermal images were recorded using Testo‐865 instruments. The periphery of the photothermal layer was wrapped with Styrofoam for heat insulation and to reduce unnecessary edge evaporation. It is worth noting that the HCAP without Styrofoam simply had the polystyrene foam removed, and all other conditions remained the same. There are three trials in every experiment to calculate the average and standard deviation.

### 4.5 Hydrovoltaic Experiments

To evaluate the electricity generation performance, two titanium plates were attached to both sides of the HCAP evaporator. The solution is added to the container so that it contacts the bottom of the sample, meaning the bottom electrode is wet while the top electrode remains dry in the air. Considering the influence of corrosion potential, titanium electrodes were chosen instead of copper electrodes. In the experiment, an electrochemical workstation (Gamry Interface 1010B) was used to record changes in current, voltage, and resistance.

## Supporting Information

Additional supporting information can be found online in the Supporting Information section.

## Author Contributions

**Xiaxia Yang**: investigation, writing – original draft, methodology, formal analysis. **Yutong Chi**: investigation, formal analysis. **Weiwei Shi**: conceptualization, writing – original draft, writing – review and editing, methodology, supervision, formal analysis.

## Conflicts of Interest

The authors declare no conflicts of interest.

## Supporting information

Supplementary Material

## Data Availability

The data that support the findings of this study are available from the corresponding author upon reasonable request.
