# Supplementary material for: High‐Performance Water Purification and Synergistic Hydrovoltaic by Avocado Peels Decorated With Carbon Dots
Source: Small Sci. 2026 Apr 20;6(4):e70284. doi: 10.1002/smsc.70284 (PMC13094410; doi:10.1002/smsc.70284)
Supplement: Supplementary file 1 — Supplementary Material [file SMSC-6-e70284-s001.pdf]

Supporting Information

High-performance Water Purification and Synergistic Hydrovoltaic by Avocado Peels Decorated with Carbon Dots

Xiaxia Yang, Yutong Chi, and Weiwei Shi*

Division of Natural and Applied Sciences, Duke Kunshan University, Kunshan, Jiangsu Province 215316, China

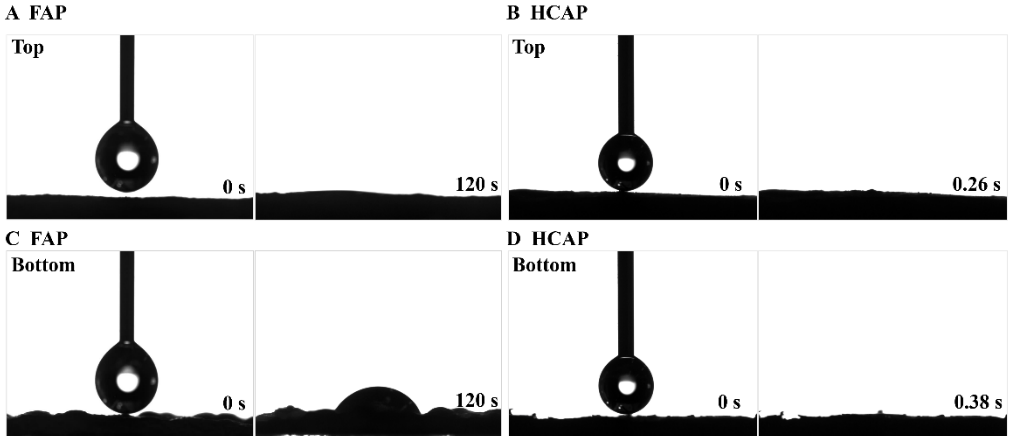

Figure S1. Contact angles of (A, C) FAP and (B, D) HCAP.

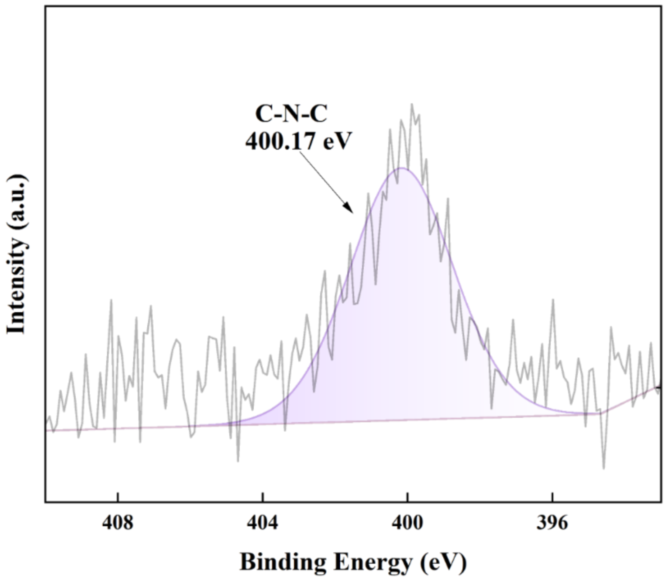

Figure S2. N1s core-level XPS spectrum of HCAP.

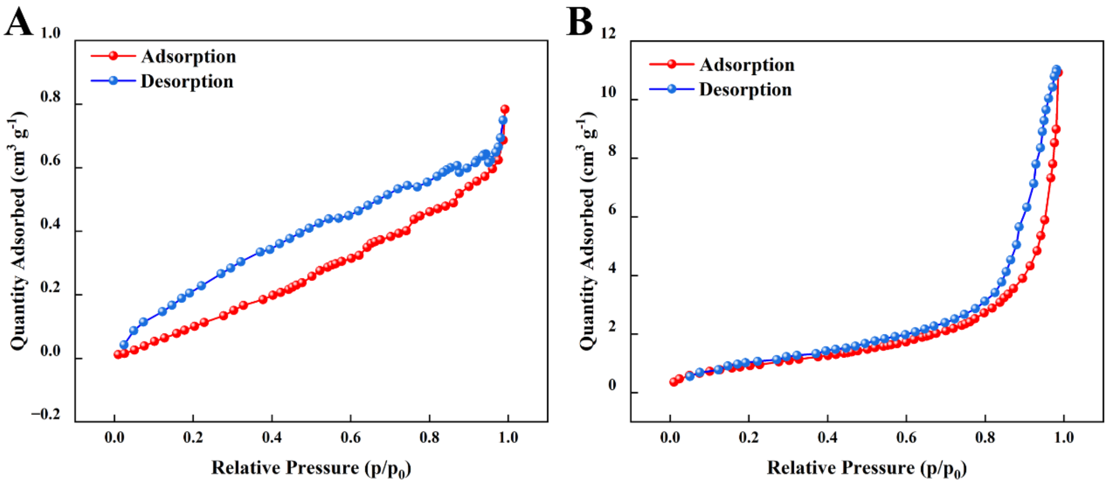

Figure S3. N_2 adsorption-desorption isotherm plots of (A) FAP and (B) HCAP.

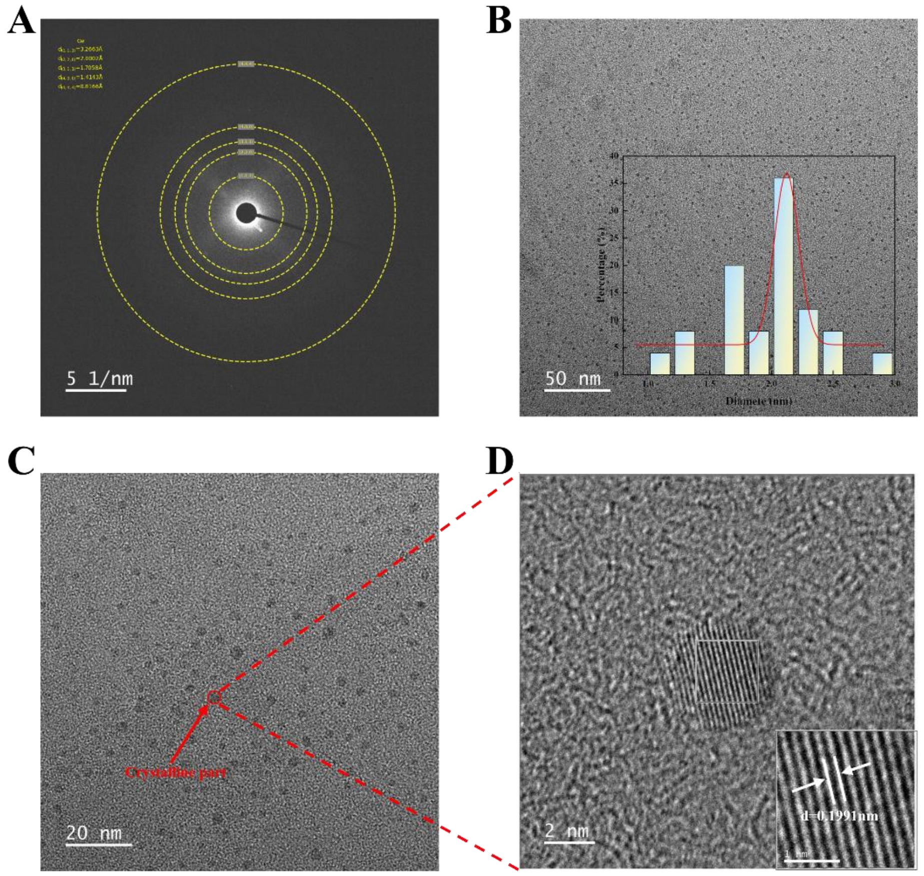

Figure S4. (A) SAED pattern of carbon dots. (B) TEM images of carbon dots with the illustration of particle size distribution histogram. (C)HR-TEM images of carbon dots that show crystalline area and (D) crystal lattice of carbon dots.

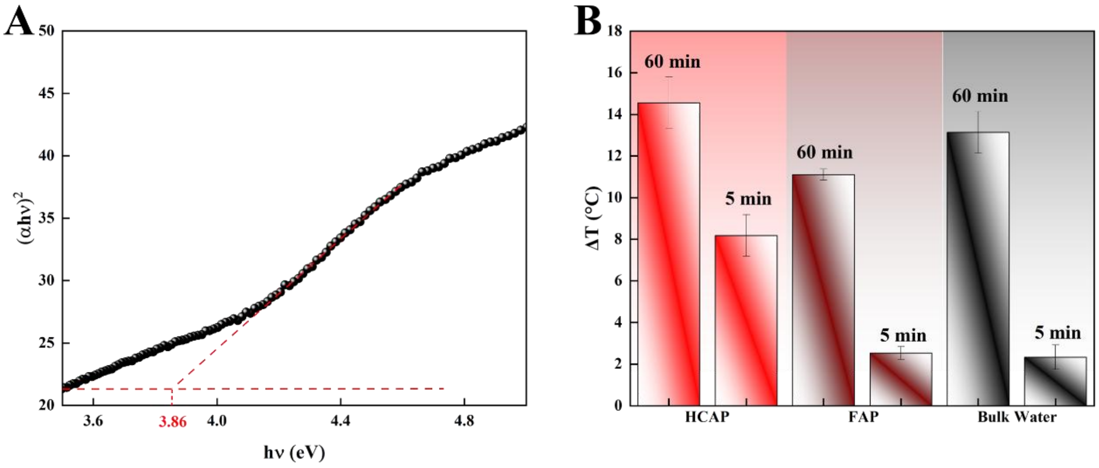

Figure S5. (A) Tauc plot of the carbon dots on HCAP. (B) The respective temperature differences of HCAP, FAP and water at 5 min and 60 min under one-sun irradiation.

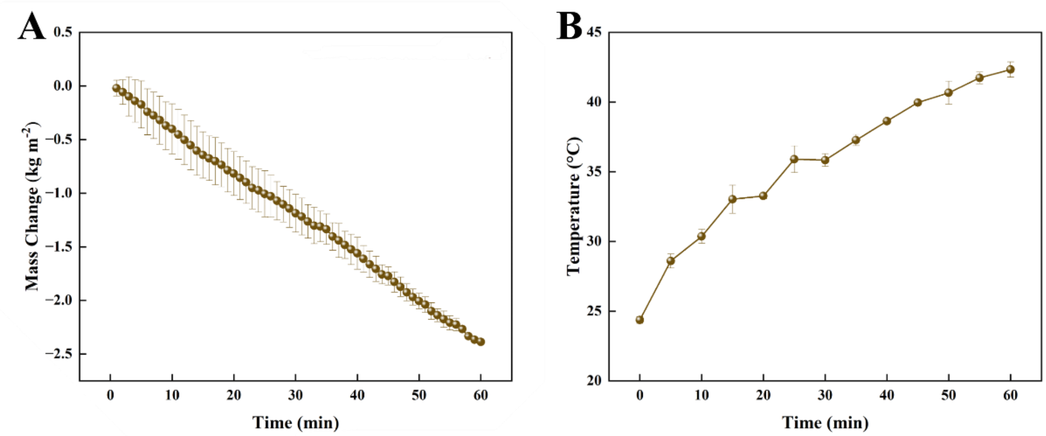

Figure S6. The solar evaporation performance of HCAP samples without Styrofoam for including (A) mass changes and (B) surface temperature under one solar irradiation.

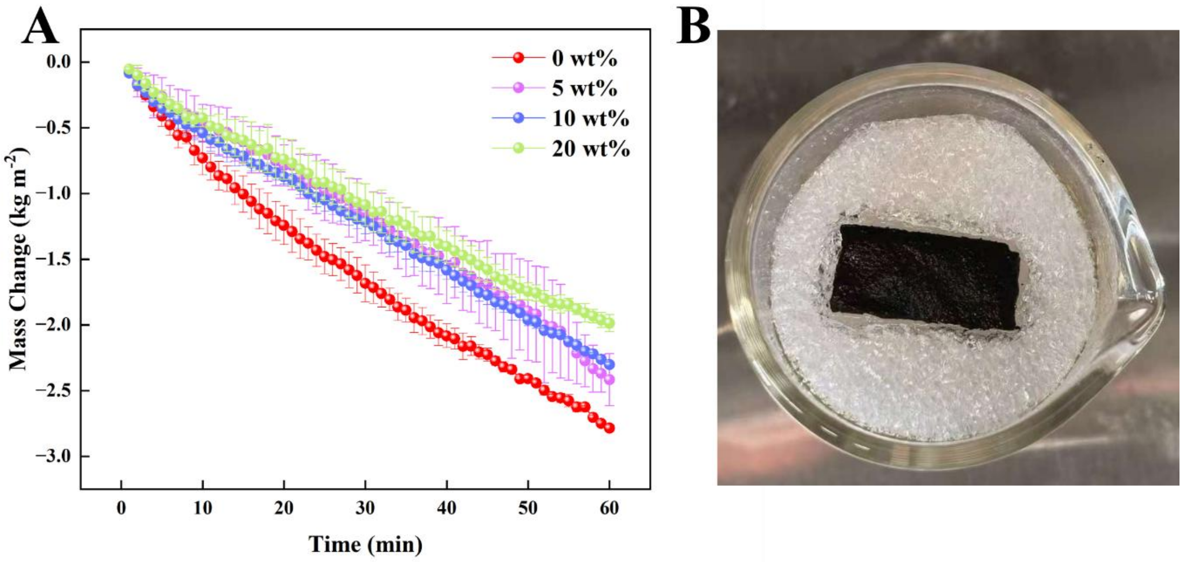

Figure S7. (A) Mass changes of HCAP under different brine water in the salinity of 0wt%, 5wt%, 10wt%, and 20wt%, respectively. (B) The optical image of HCAP after 8-cycle water desalination process.

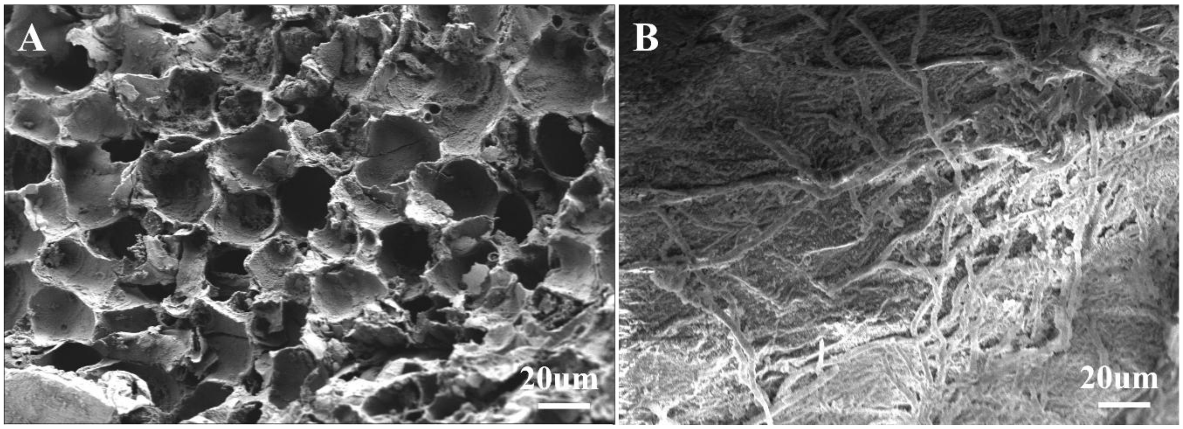

Figure S8. SEM images after the 8 cycles at (A) the top surface, and (B) the bottom surface of HCAP.

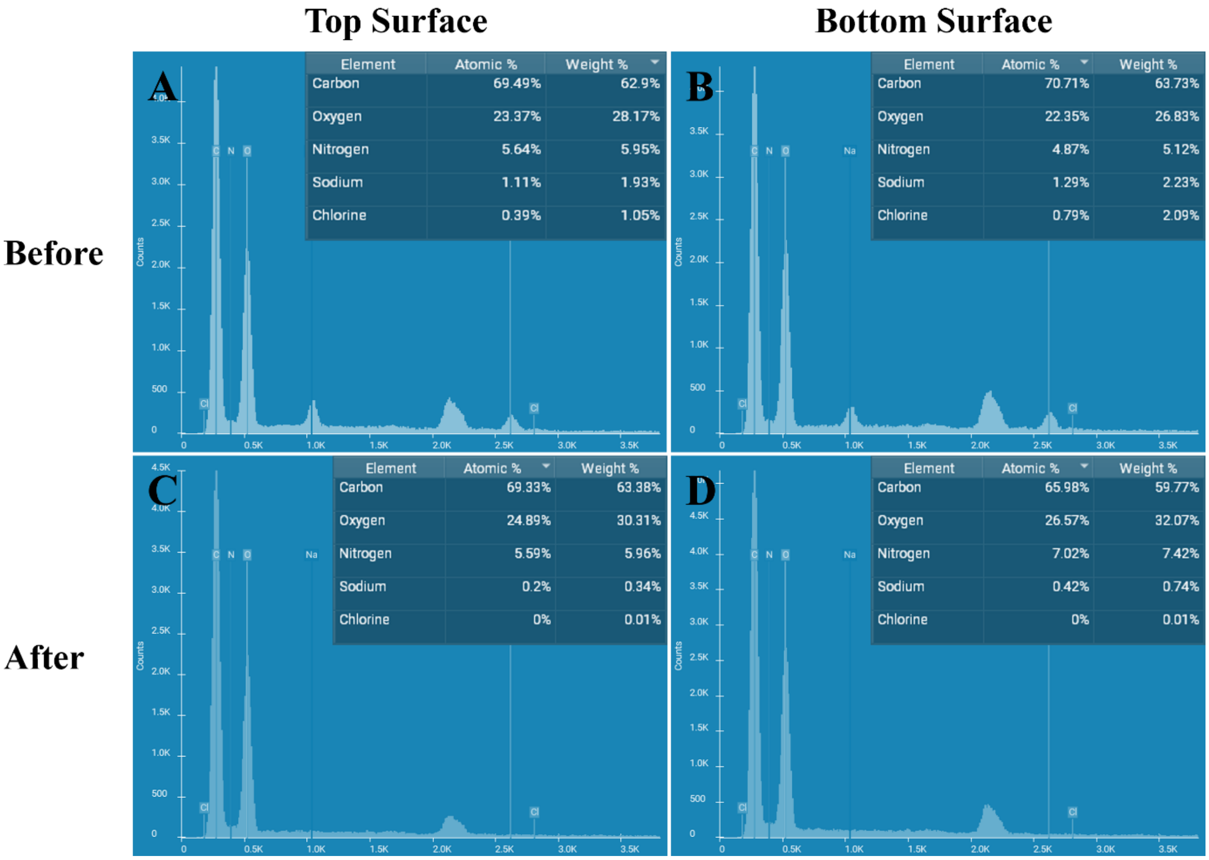

Figure S9. EDS elemental analysis on HCAP before 8 cycles at (A) the top surface and (B) the bottom surface, and after 8 cycles at (C) the top surface and (D) the bottom surface.

Supporting Information Note 1

The photothermal conversion efficiency ($\eta - \mathrm{PCE}$)

The photothermal efficiency, referring to how much energy is transformed from light to heat, determined by the intrinsic properties and architecture of photothermal materials[1-2],

$$(\boldsymbol{\eta} - \mathbf{PCE}) = \frac{\mathbf{P_{evaporation}}}{\mathbf{P_{light}}} \times \mathbf{100}$$

$$\boldsymbol{P_{light} = \alpha C_{opt} q_i}$$

$$\boldsymbol{P_{evap} = P_{light} - P_{env}}$$

Where, P_{env} is the power density loss during evaporation through radiation and convection

$$\boldsymbol{P_{env}} = \mathbf{P_{radiative} + P_{convective}}$$

$$\boldsymbol{P_{env} = \varepsilon\sigma\,(T_2^4 - T_1^4) + h\,(T_2 - T_1)}$$

Optical absorption coefficient, $\alpha = 0.95$

Optical concentration, $C_{opt} = 1$

Emissivity of the material, $\varepsilon = 0.93$

Stefan-Boltzmann constant, $\sigma = 5.67 \times 10^{-8}$ W m^{-2} K

Solar flux, $q_i = 1000$ Wm^{-2}

Convection heat transfer coefficient, h = 5 W m^{-2} K^{-1}

Temperature at the adjacent environment, after illumination $T_1 = 303.15$ K

Temperature at the evaporating surface after illumination $T_2 = 315.15$ K

$P_{radiative} = \varepsilon\sigma\,(T_2^4 - T_1^4) = 73.16$ W m^{-2}

Radiative loss (%) = $\frac{\text{P radiative}}{\text{P light}} \times 100 = \frac{73.16}{950} = 7.7$ %

$P_{convective} = h\,(T_2 - T_1) = 60$ W m^{-2}

Convective loss (%) = $\frac{\text{Pconvective}}{\text{Plight}} \times 100 = \frac{60}{950} = 6.32$ %

$P_{env} = 133.16$ W m^{-2}

$P_{evap} = 816.84$ W m^{-2}

η-PCE = $\frac{816.84}{950} = 86\%$

Supporting Information Note 2

The water evaporation efficiency(η-WEE)

The water evaporation efficiency of an evaporator under a solar simulator is calculated, which refers to how much transformed heat is used in evaporation[3-4]:

$$\eta - WEE = \frac{\dot{m} h_{lv}}{C_{opt} q_{in}}$$

where $\dot{m}$ is the net evaporation rate (equal to the actual evaporation rate minus 0.323 kg m^{-2} h^{-1}, the average evaporation rate in the dark), C_{opt} is the optical concentration, and q_{in} denotes the incident solar irradiation power (kW m^{-2}), and h_{lv} represents the latent enthalpy of the liquid-vapor phase change of water (kJ kg^{-1}), which can be estimated as:

$$h_{lv} = \lambda_{lv} + C_p \Delta T$$

where λ_{lv} is the latent heat of water evaporation under the standard atmospheric pressure (2.257 MJ kg^{-1}), C_{P} represents the specific heat capacity of bulk water (4.2 kJ kg^{-1} K^{-1}), and ΔT is the temperature variation during the evaporation process.

Supporting Information Note 3

The energy use pathway methods

The heat transfer process of solar vapor generation includes three energy flows[5]: solar energy input, vapor output, and heat exchange with the environment, in the form of either energy gain from environment or energy loss to environment.

$$\boldsymbol{P_{solar} + Q_{env} = Q_e + Q_r + Q_c + Q_{cond}}$$

Where P_{solar} is the incident solar power, Q_{env} is the environmental heat gain, Q_e is the energy utilized for evaporation (latent heat), Q_r and Q_c are the radiative and convective heat losses to the surroundings, and Q_{cond} is the conductive heat loss to the bulk water.

The detailed breakdown is summarized as follows:

Evaporation utilization (Q_e): Based on the measured evaporation rate of 2.79 $kg \cdot m^{-2} \cdot h^{-1}$ and the equivalent latent heat (2257 $J \cdot g^{-1}$), Q_e is calculated to be ≈1749 $W \cdot m^{-2}$.

Environmental heat gain (Q_{env}): During the steady-state evaporation, the side temperature of the cylindrical system (≈21°C) was consistently lower than the ambient temperature (30°C). This temperature gradient drives a heat influx from the environment, can be described as

$$\boldsymbol{Q_{env} = h \times \left(\frac{A_{side}}{A_{top}}\right) \times (T_a - T_{side})}$$

Where Q_{env} is the environmental heat gain, h is the convective coefficient (5 $W \cdot m^{-2} \cdot K^{-1}$), A_{side} represents the lateral surface area ($81 cm^2$), and A_{top} denotes the top surface area of the evaporator ($4 cm^2$), T_a (30°C) denotes the ambient temperature, and T_{side} (21°C) denotes the lateral surface temperature of the evaporator. Q_{env} is calculated to be ≈911 $W \cdot m^{-2}$

Radiation heat losses (Q_r): The radiation heat loss per unit area can be calculated using the Stefan-Boltzmann law:

$$\mathbf{Q_r = \varepsilon \boldsymbol{A_{top}} \sigma \left(T_s^4 - T_a^4\right)}$$

Where ε is the infrared emissivity of the evaporator (0.93); σ is the Stefan-Boltzmann constant (5.67×10^{-8} W m^{-2} K^{-4}); T_a is the adjacent environment temperature (30℃); and T_s denotes the mean top surface temperature of the evaporator (42℃);).Thus, Q_r≈73.16 W·m^{-2}

Convection heat loss (Q_c): Convection heat loss per unit area can be calculated using the Newton's cooling equation:

$$Q_c = A_{top} h (T_s - T_a)$$

Thus, Q_c≈60 W·m^{-2}

Conduction heat loss (Q_{cond}): The use of the Styrofoam insulator effectively minimized the heat transfer to the bulk water, rendering Q_{cond} negligible.

Supporting Information Note 4

The salt rejection ratio (R)

The salt rejection ratio (R) was estimated according to the formula[6]:

$$\boldsymbol{R} = \left(\mathbf{1} - \frac{\boldsymbol{C_p}}{\boldsymbol{C_f}}\right) \times \mathbf{100}\%$$

Where C_p and C_f represent the salt concentrations of the collected water and the seawater, respectively. The salt concentrations were obtained by ICP-MS testing.

References

[1] S. L. Wu, H. L. Chen, H. L. Wang, X. L. Chen, H. C. Yang, S. B. Darling, *ENVIRONMENTAL SCIENCE-WATER RESEARCH & TECHNOLOGY* **2021**, 7, 24.

[2] M. A. Villan, A. Suresh, M. Misra, C. N. Ruiz, N. R. Cameron, S. K. Saha, S. Chandramouli, *ADVANCED SCIENCE* **2026**, 13.

[3] X. Yang, X. Yang, T. Lu, W. Shi, *ACS Applied Engineering Materials* **2025**, 3, 1579.

[4] H. X. Liang, Y. L. Mu, M. Y. Yin, P. P. He, W. W. Guo, *SCIENCE ADVANCES* **2023**, 9.

[5] J. H. Zhao, Y. H. Wu, M. Xia, J. H. Yu, D. Wang, L. Lv, J. X. Xiao, *CHEMICAL ENGINEERING JOURNAL* **2026**, 531.

[6] K. Takeuchi, Y. Takizawa, H. Kitazawa, M. Fujii, K. Hosaka, J. Ortiz-Medina, A. Morelos-Gomez, R. Cruz-Silva, M. Fujishige, N. Akuzawa, M. Endo, *DESALINATION* **2018**, 443, 165.
